# Pancreatic metastasis from invasive pleomorphic lobular carcinoma of the breast: a rare case report

**DOI:** 10.1186/s13000-017-0641-4

**Published:** 2017-07-11

**Authors:** Xiangjie Sun, Ke Zuo, Dan Huang, Baohua Yu, Yufan Cheng, Wentao Yang

**Affiliations:** 1 Department of Pathology, Fudan University Shanghai Cancer Center, No.2 building, 270 Dong’an Road, Shanghai, 200032 China; 2 Department of Oncology, Shanghai Medical College, Fudan University, Shanghai, 200032 China

**Keywords:** Pleomorphic lobular carcinoma, Triple-negative breast cancer, GATA3, Metastasis

## Abstract

**Background:**

Invasive pleomorphic lobular carcinoma (PLC) is an aggressive subtype of invasive lobular carcinoma of the breast, which has its own histopathological and biological features. The metastatic patterns for PLC are distinct from those of invasive ductal carcinoma. In addition, pancreatic metastasis from PLC is extremely rare.

**Case presentation:**

We report a rare case of a 48-year-old woman presenting with clinical gastrointestinal symptoms and pancreatic metastasis of PLC. The pancreatic tumor was composed of pleomorphic tumor cells arranged in the form of solid sheets and nests and as single files, with frequent mitotic figures, nucleolar prominence, high nuclear to cytoplasmic ratio and loss of cohesion. The malignant cells were positive for p120 (cytoplasmic) and GATA3 and negative for estrogen receptor, progesterone receptor, human epidermal growth factor receptor 2, E-cadherin, gross cystic disease fluid protein 15 and mammaglobin, which indicated a lobular carcinoma phenotype of the breast.

**Conclusions:**

To the best of our knowledge, this is one of the few reported cases in the literature of pancreatic metastasis of invasive lobular carcinoma of the breast, of which the definitive diagnosis was obtained only after surgery. Rare metastasis sites should be considered, particularly, when a patient has a medical history of PLC.

## Background

Invasive lobular carcinoma (ILC) of the breast is the second most common type of invasive breast carcinoma, representing 8–14% of all breast carcinomas [1]. ILC can be divided into classic, alveolar, solid, tubulolobular, pleomorphic and mixed subtypes according to histological features [2].It is commonly accepted that invasive pleomorphic lobular carcinoma (PLC) is associated with aggressive tumor behavior and poor clinical outcomes. The metastatic patterns of ILC are distinct from those of invasive ductal carcinoma (IDC), and metastasis to uncommon sites may occur including gastrointestinal or peritoneal metastasis [3].

## Case presentation

This case study was approved by the Institutional Review Board of the ethical committee of Fudan University Shanghai Cancer Center.

**Fig. 1:**
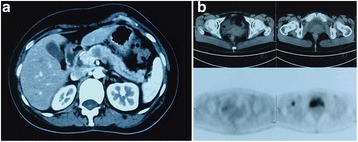
**a**: Contrast-enhanced computed tomography scan of the abdomen showing a low-density mass confined to the neck of pancreas. **b**: Positron emission tomography/computed tomography scan of the pelvis: a lytic bony lesion in the right caput femoris, with abnormal uptake of fluorodeoxyglucose

A 48-year-old woman presented with epigastric discomfort for several weeks, and the levels of serum tumor markers were normal. Computed tomography (CT) of the abdomen showed a mass (diameter 21.7 mm) confined to the neck of the pancreas with low density and two enlarged lymph nodes approximately 8 mm in size around the pancreas (Fig. 1a). Subsequently, she underwent positron emission tomography/computed tomography (PET/CT). A lytic bony lesion in the caput femoris was found besides the pancreatic mass (Fig. 1b), while there were no signs of metastasis in the axillary and supraclavicular lymph nodes, liver or lung. After consideration of imaging manifestations of malignancy, the patient underwent distal pancreatosplenectomy.

**Fig. 2:**
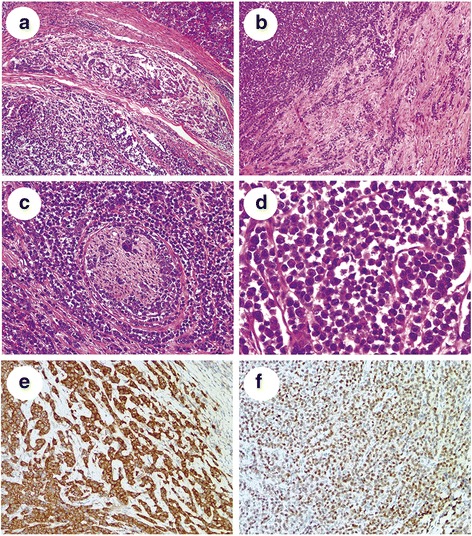
Photomicrograph of the lesion in pancreas. **a**: The malignant cells infiltrating the lobules of pancreas (H&E, ×100). **b**: The tumor cells arranging in the form of solid sheets and single files (H&E, ×100). **c**: The tumor cells demonstrating a targetoid growth pattern and nerve invasion (H&E, ×200). **d**: High magnification showing the polygonal cell contours, abundant eosinophilic cytoplasm, high nuclear to cytoplasmic ratio and loss of cohesion (H&E, ×400). **e**: Diffuse strong cytoplasmic staining for p120 (Envision, ×200). **f**: Diffuse strong nuclear staining for GATA3 (Envision, ×200)

Microscopic analysis revealed that the tumor was composed of pleomorphic cells infiltrating the lobules of the pancreas (Fig. 2a). The malignant cells were arranged in the form of solid sheets and nests and as single files, with high nuclear to cytoplasmic ratio and loss of cohesion (Fig. 2b, c). The cell contours were round to polygonal. The cytoplasm of the cells was abundant and eosinophilic, with nuclei of increased size, frequent mitotic figures, and nucleolar prominence (Fig. 2d). There were no metastases in any of the ten lymph nodes removed around the pancreas.

Immunohistochemical staining revealed that the tumor cells were positive for p120 (cytoplasmic) and GATA3 (Fig. 2e, f) and negative for estrogen receptor (ER), progesterone receptor (PR), human epidermal growth factor receptor 2 (HER2), E-cadherin, gross cystic disease fluid protein 15 (GCDFP-15) and mammaglobin.

**Fig. 3:**
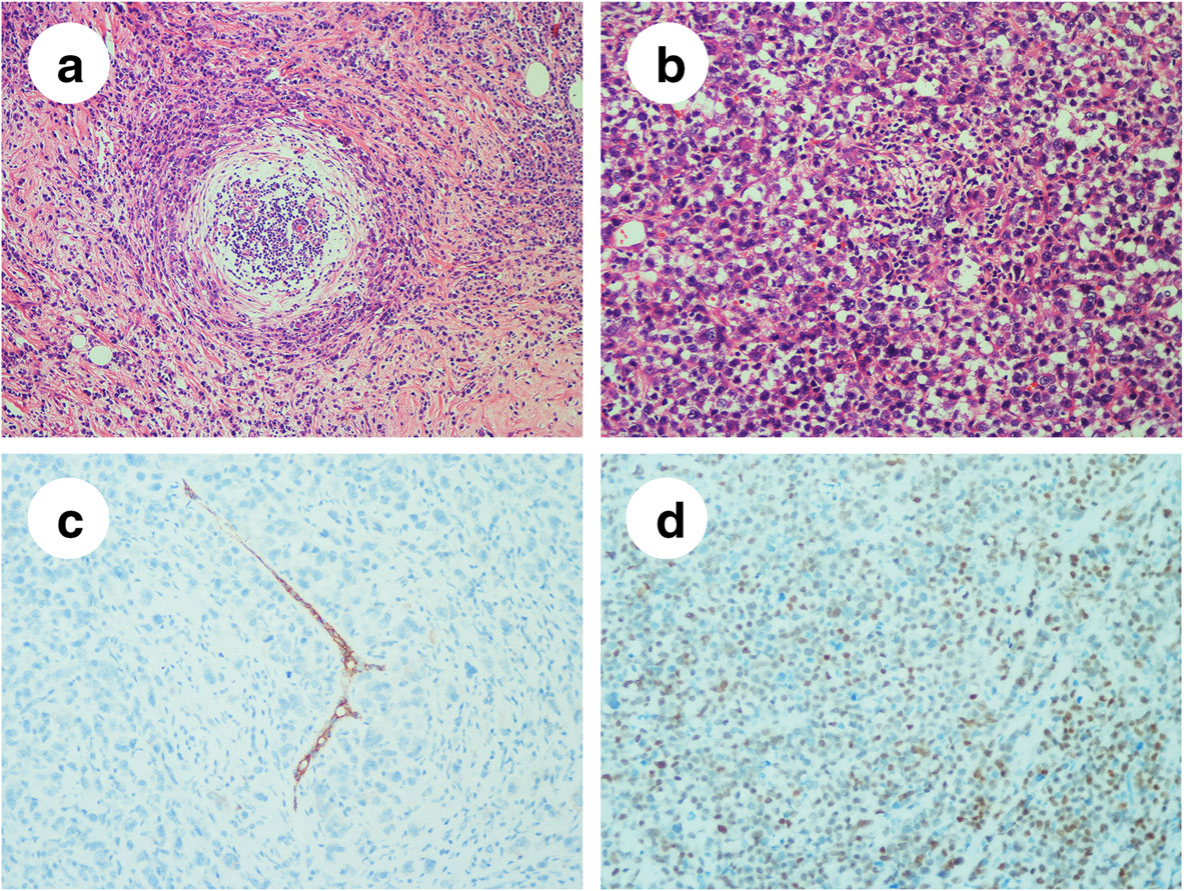
Photomicrograph of the primary breast lesion. **a**: The malignant tumor cells showing a targetoid growth pattern or arranging in the form of solid sheets and single files (H&E, ×100). **b**: Abundant and eosinophilic cytoplasm of the cells, with nuclei of increased size and nucleolar prominence (H&E, ×200). **c**: Negative E-cadherin immunostaining of tumor cells with positive internal control in the residual duct (Envision, ×200). **d**: Moderate to strong nuclear staining for GATA3 (Envision, ×200)

Past medical history of this patient was significant for a modified radical mastectomy. Two years ago, a lump was found in her right breast without swelling or pain, and she chose to follow up. The patient presented to our outpatient clinic because of dragging pain in her right breast 10 months later. Physical examination revealed a breast mass (approximately 4 × 4 cm) in the internal inferior quadrant, and invasive carcinoma was confirmed by core needle biopsy. Ultrasonography showed no other metastases in the left breast, supraclavicular lymph nodes or abdomen. The patient was diagnosed with invasive PLC (4.5 × 3.5 × 2.0 cm in size) after modified radical mastectomy (Fig. 3a, b), with the absence of metastases in all 17 lymph nodes removed (pT2 N0 M0, stage IIA). By immunohistochemistry, the tumor showed a triple-negative breast cancer (TNBC) phenotype (ER−/PR−/HER2-), negative for E-cadherin (Fig. 3c), positive for GATA-3 (Fig. 3d), and the Ki-67 index was approximately 40%. After mastectomy, she received adjuvant chemotherapy with paclitaxel for 6 courses and then underwent regular follow up.

The profile of this case is in accordance with the diagnosis of metastatic pleomorphic lobular breast cancer in the pancreas. The patient was then treated with capecitabine, an orally administered chemotherapeutic agent used in the treatment of numerous cancers, including metastatic breast cancer.

## Discussion

As a variant of ILC, invasive PLC is rare, accounting for less than 1% of all epithelial malignancies of the breast, and associated with older age and postmenopausal status [4–6]. It remains to be determined whether the pleomorphic histology of PLC independently predicts a worse prognosis. Compared to classic ILC, invasive PLC is known to be associated with larger tumors, more frequent lymphovascular invasion, increased lymph node involvement, increased incidence of TNBC, and increased likelihood of distant metastases, which are all unfavorable prognostic factors in breast cancer [7, 8].

Unlike most classical ILC demonstrating luminal subtype, ER and PR expression can be both lost in PLC, with PR loss observed in one-third of cases in a majority of studies, and HER2 amplification being found in approximately one-quarter of PLC cases [8]. Compared to IDC, loss of membranous expression for E-cadherin is a defining feature in most ILC cases. A few studies have indicated that loss of E-cadherin expression can be found in 80% to 100% of PLC cases [9]. When E-cadherin is dysfunctional, p120, as a member of the E-cadherin complex, accumulates in the cytoplasm, which in turn sets into motion a chemical cascade to increase cell motility [10]. The utilization of both E-cadherin and p120 as markers has been considered the best diagnostic strategy in distinguishing lobular from ductal carcinomas of the breast.

The metastatic pattern for PLC is distinct, the most common sites of which are bone and liver, followed by lung, peritoneum, skin, chest wall and central nervous system [7, 11]. Pancreatic metastasis from other cancer is rare. Renal cell carcinoma is the most common tumor metastasizing to pancreas, while breast cancer metastasis to pancreas is extremely rare, which forms only a minority of an already small patient population. The majority of the literature devoted to the diagnosis and treatment of these patients consists of anecdotal case reports [12–14]. Previously published series have demonstrated that metastatic involvement of the pancreas from primary breast cancer as solitary metastasis sites has an incidence lower than 3% [14]. And almost all the cases that described the pancreatic metastasis were ILC, fewer cases were IDC, none of these was presented with PLC [14–16].Therefore, our report may have the particular meaning in consideration of the differential diagnosis of pancreatic metastasis in case that the prior history is unknown.

It has been reported that ILC metastasizes to organs such as the pancreas, uterus, gastrointestinal tract, urinary tract, reproductive organs and the retroperitoneum after a long disease-free interval [17].In contrast, our patient with PLC had a fairly rapid recurrence, highlighting the potential significant difference in the underlying biology of these two tumors.

Additionally, ILC is seen more often than IDC in cases of breast cancer that is metastatic to the abdominal organs, such as pancreas and gastrointestinal tract [14].The reasons for the different metastatic patterns between ILC and IDC are poorly understood. The loss of expression of E-cadherin in ILC is one possible explanation [18]. It has been demonstrated that loss of expression of E-cadherin in ILC may decrease adhesiveness of cells and facilitate the infiltration through connective tissues and over peritoneal surfaces [19, 20]. Besides that, in some certain areas with microanatomy, it is more conducive to stop or trap some types of cells with special size or shape. Moreover, the microenvironment of the ovary or peritoneum may provide growth and survival factors that favor ILC cells over IDC cells [19]. The list of anatomical sites associated with ILC metastasis, e.g. ovaries, abdominal cavity, skin and bone, seems like a catalog of tissue compartments with relatively more steroid hormone supply. As compared with the body circulation, estrogen concentrations are much more higher in ovarian tissue and peritoneal cavity fluid [21]. Despite all this,the mechanism involved in ILC metastasis still needs further exploration.

It is difficult to diagnosis metastasis when it occurs in a rare site. Histological characterization is crucial, since the features of PLC are extraordinary. Similar to classic ILC, PLC usually consists of diffuse infiltration of discohesive tumor cells arranged in single files or sheets, and it frequently demonstrates a targetoid growth pattern around terminal ducts and lobules. The nucleus of PLC cells can be nearly four times the size of that of a lymphocyte, while the nucleus of classic ILC cells tends to be only 1–2 times the size of a lymphocyte nucleus [11, 22].

Immunohistochemistry is a reliable method to distinguish between a primary and metastatic tumor. Currently, GATA3 is the most sensitive marker of mammary differentiation, particularly in the setting of metastatic TNBCs, which are typically negative for other traditional mammary-specific markers such as mammaglobin and GCDFP-15. GATA3 positivity has been reported in 73–96% cases of metastatic breast carcinoma (MBC) [23]. The frequency of GATA3, mammaglobin, and GCDFP-15 was 95%, 78%, and 65%, respectively, in a study consisting of MBC cases (*n* = 166) [24]. GATA3 may have the greatest diagnostic potential in TNBCs. As reported, GATA3 was observed in 44% to 66%, mammaglobin in 26%, and GCDFP-15 in only 16% of primary TNBC cases by immunohistochemistry (*n* = 111) [25]. In this case, the primary tumor in the right breast was triple-negative, and the metastatic lesion in pancreas was negative for ER, PR, HER2, mammaglobin and GCDFP-15 but showed diffuse strong staining for GATA3.

Nevertheless, we should pay more attention to the specificity of GATA3 when it is used to distinguish primary pancreatic tumors and metastasis from breast. According to the research of Miettinen et al., besides the strong nuclear GATA3 positivity in primary and metastatic carcinomas of the breast, GATA3 was also expressed in a wide range of tissues and tumors, including pancreatic ductal carcinoma, but in a relative lower frequency (37%) [26]. Therefore, in differentiating a pancreatic ductal carcinoma from metastatic breast cancer, it is important to use a panel of antibodies instead of one or two antibodies. However, in this case, the diagnosis is straightforward because of the clinical history and special morphology of the tumor cells.

For a pancreatic mass with tumor cells arranged in the form of solid sheets or nests, pancreatic acinar cell carcinoma, pancreatic neuroendocrine tumor, pancreatoblastoma and solid-pseudopapillary neoplasm of the pancreas should also be considered in differential diagnoses. Clinical features, imaging examinations and immunohistochemical tests assist in accurate diagnosis. Additionally, neuroendocrine tumors in pancreas, including low-grade tumors, such as carcinoids and well-differentiated pancreatic neuroendocrine tumors were negative for GATA3 [26].

Considering the specific management of PLC, there are no guidelines yet. Aggressive managements are expected due to the adverse histopathological and biological features and poorer outcomes. It is accepted that patients with PLC are more likely to require mastectomy than patients with classic ILC, and currently there is a tendency to offer adjuvant chemotherapy for PLC [5, 7, 8]. For metastatic lesions, surgical intervention does not significantly extend overall survival but may be considered as a palliative treatment [3, 27].

## Conclusion

The understanding of PLC has progressed dramatically in the past decade. Distal metastasis should be taken into account when a patient has a medical history of invasive PLC, even when a rare metastasis site is involved. Histopathological characteristics and immunohistochemical tests are helpful for diagnosis.

## Abbreviations

CT: computed tomography
ER: estrogen receptor
GCDFP-15: gross cystic disease fluid protein 15
HER2: human epidermal growth factor receptor 2
IDC: invasive ductal carcinoma
ILC: invasive lobular carcinoma
PET/CT: positron emission tomography/computed tomography (PET/CT)
PLC: pleomorphic lobular carcinoma
PR: progesterone receptor
TNBC: triple-negative breast cancer
